# Evident bacterial community changes but only slight degradation when polluted with pyrene in a red soil

**DOI:** 10.3389/fmicb.2015.00022

**Published:** 2015-01-30

**Authors:** Gaidi Ren, Wenjie Ren, Ying Teng, Zhengao Li

**Affiliations:** Key Laboratory of Soil Environment and Pollution Remediation, Institute of Soil Science – Chinese Academy of SciencesNanjing, China

**Keywords:** PAH pollution, pyrene, biodegradation, bacterial community, 16S rRNA gene, Illumina sequencing, red soil

## Abstract

Understanding the potential for Polycyclic aromatic hydrocarbons (PAH) degradation by indigenous microbiota and the influence of PAHs on native microbial communities is of great importance for bioremediation and ecological evaluation. Various studies have focused on the bacterial communities in the environment where obvious PAH degradation was observed, little is known about the microbiota in the soil where poor degradation was observed. Soil microcosms were constructed with a red soil by supplementation with a high-molecular-weight PAH (pyrene) at three dosages (5, 30, and 70 mg ⋅ kg^-1^). Real-time PCR was used to evaluate the changes in bacterial abundance and pyrene dioxygenase gene (*nidA*) quantity. Illumina sequencing was used to investigate changes in diversity, structure, and composition of bacterial communities. After 42 days of incubation, no evident degradation was observed. The poor degradation ability was associated with the stability or significant decrease of abundance of the *nidA* gene. Although the abundance of the bacterial 16S rRNA gene was not affected by pyrene, the bacterial richness and diversity were decreased with increasing dosage of pyrene and the community structure was changed. Phylotypes affected by pyrene were comprehensively surveyed: (1) at the high taxonomic level, seven of the abundant phyla/classes (relative abundance >1.0%) including Chloroflexi, AD3, WPS-2, GAL5, Alphaproteobacteria, Actinobacteria, and Deltaproteobacteria and one rare phylum Crenarchaeota were significantly decreased by at least one dosage of pyrene, while three phyla/classes (Acidobacteria, Betaproteobacteria, and Gammaproteobacteria) were significantly increased; and (2) at the lower taxonomic level, the relative abundances of twelve orders were significantly depressed, whereas those of nine orders were significantly increased. This work enhanced our understanding of the biodegradation potential of pyrene in red soil and the effect of pyrene on soil ecosystems at the microbial community level.

## INTRODUCTION

Polycyclic aromatic hydrocarbons (PAHs) composed of two or more fused aromatic rings are one of the most prevalent groups of organic contaminants in soil. Due to their persistence and toxicity, PAHs have become a substantial threat to the stability and function of soil ecosystems. Increased anthropogenic activities such as the combustion of wood, coal and petroleum and mining accidents have dramatically exacerbated soil pollution with PAHs. Microorganisms play a critical role in PAH transformation and degradation in terrestrial ecosystems because microbial degradation is the principal process underlying natural decontamination (Viñas et al., 2005). Under conditions of long-term PAH pollution, some native adapted microorganism may be able to utilize the bio-available PAHs as their sole carbon or energy source, resulting in the degradation of PAHs *in situ*. Some PAH-degradative populations have been isolated and characterized using culture-dependent methods (Heitkamp et al., 1988; Masakorala et al., 2013; Ping et al., 2014; Yuan et al., 2014); these populations are frequently affiliated with a limited number of taxonomic groups such as *Sphingomonas*, *Burkholderia*, *Pseudomonas*, *Rhodococcus*, and *Mycobacterium* (Uyttebroek et al., 2006). Molecular ecological approaches have also been developed to study PAH-degradative bacterial populations. For example, a previous study (DeBruyn et al., 2009) evaluated the potential for PAH biodegradation by indigenous microbial communities through the detection and quantification of a key PAH-degradative gene (pyrene dioxygenase gene: *nidA*). The authors found that pyrene-degrading *Mycobacterium* showed a broad geographical distribution and may play an important role in the natural attenuation and cycling of PAHs in Lake Erie.

The biodegradation of PAHs is influenced by several biotic (Sipilä et al., 2008) and abiotic (Simarro et al., 2011) factors. The type of PAH (Singleton et al., 2005; DeBruyn et al., 2011), plant exudate (Sipilä et al., 2008) and size fractions of the soil (Uyttebroek et al., 2006) have been shown to influence the composition and abundance of total or degradative bacterial populations in polluted soil. Therefore, PAHs may not always be degraded by native microorganisms, especially in soils that have not experienced historical PAH contamination, although there are reports of PAH degradation in soils without historical PAH contamination (Tervahauta et al., 2009; Yrjälä et al., 2010). The microbial populations in polluted soil or sediments have been well studied, but the PAH degradation potential exhibited by indigenous microbiota and microbial community changes in unpolluted soils threatened with PAH pollution are not well understood.

Several possibilities may occur when unpolluted soil suffers from PAH pollution. Some microbial populations may adapt rapidly to pollution and use the PAHs as carbon or energy sources for survival, leading to degradation. In contrast, the PAHs may exert toxic effects on the microorganisms where biodegradation does not occur, resulting in the loss of microbial diversity. Some populations may again not suffer from toxic effects, but are also not degraders of PAHs (Mukherjee et al., 2013, 2014). Various studies have focused on the PAH degrading bacteria and the total bacterial communities in the environments where obvious degradation was observed (Peng et al., 2010; Singleton et al., 2013; Sauret et al., 2014; Sun et al., 2014), little is known about the relationship between the degradation and the PAH degrading bacterial populations and influence of PAH on the microbial community in the case where PAH degradation did not happen. Red soils are widely distributed in China, covering more than 20% of the country’s total land area and constituting one of the most important soil sources for food production (Huang and Zhao, 2014). Understanding the relationship between PAH degradation potential and the associated microbial community changes is important for ecological evaluation of the effect of PAHs on soil ecosystems from the viewpoint of microbial ecology. Furthermore, increased understanding is a prerequisite for directing the management and cleanup of PAH-contaminated soil ecosystems.

Soil is considered to be a highly complex system with an overwhelming diversity of bacterial communities. It is estimated that a typical gram of soil contains one billion bacterial cells and thousands to millions of bacterial species (Gans et al., 2005). Therefore, some conventional molecular biology approaches such as terminal restriction fragment length polymorphism (T-RFLP), denaturing gel gradient electrophoresis (DGGE), and 16S rRNA-based clone library construction may not be sensitive enough to resolve the changes caused by PAH pollution at the microbial community level because of the lower resolution of these techniques. The advent of next generation sequencing techniques have opened new frontiers in microbial community analysis by providing unprecedented levels of coverage and resolution of the microbial community because this technique can generate millions to billions of sequence reads in a single run (Huse et al., 2008; Hollister et al., 2010). These techniques have been used to identify the bacterial populations that are correlated with PAH degradation (Singleton et al., 2011; Kawasaki et al., 2012; Sun et al., 2014), evaluate the bacterial community changes during the bioremediation process of PAH polluted soil (Kawasaki et al., 2012; Singleton et al., 2013), assess the association between the spatial patterns of bacterial diversity and the distribution of pollutants [including PAHs and total hydrocarbons (C10–C40)] (Mukherjee et al., 2014), as well as to determine the complete genomic sequences of PAH degradation strains (Jin et al., 2011; Zhang et al., 2012).

In this study, microcosm incubation experiment was performed with an unpolluted red soil by supplementation with a high-molecular-weight PAH (pyrene). Illumina sequencing and real-time PCR techniques were used to investigate the bacterial community changes. Of particular interest are (1) whether the native bacterial community can adjust in a short time leading to the degradation of pyrene, (2) how the bacterial community is adjusting to additions of pyrene to clean soil, and (3) how the fate of added pyrene can be explained by combining bacterial community analysis and real-time PCR of the *nidA* gene.

## MATERIALS AND METHODS

### SITE DESCRIPTION AND SOIL SAMPLING

The red soil was collected from surface soil at a depth of 0–15 cm from a long-term field experiment station in Yingtan, Jiangxi province, China (28° 15′30^′′^ N, 116° 55′30^′′^E). The soil was passed through a 2 mm mesh to remove stones and plant debris and stored at 4°C before the batch experiment. The soil properties were as follows: pH (H_2_O) 4.51, organic matter 6.60 g ⋅ kg^-1^, total N 0.39 g ⋅ kg^-1^, total P 0.015 g ⋅ kg^-1^, total K 8.54 g ⋅ kg^-1^, available N 40.8 mg ⋅ kg^-1^, available P 1.33 mg ⋅ kg^-1^, available K 47.0 mg ⋅ kg^-1^, and cation exchange capacity (CEC) 9.06 cmol.kg^-1^. The initial total concentration of 16 USEPA PAHs (ΣPAHs) was 38.4 μg ⋅ kg^-1^ soil (on dry weight soil, i.e., *d.w.s.*); the initial concentration of pyrene was 10.3 μg ⋅ kg^-1^ *d.w.s.*. This indicated that the tested soil was not polluted with PAHs as the soils with content of ΣPAHs <200 μg ⋅ kg^-1^ are classified as non-contaminated soils (Maliszewska-Kordybach, 1996).

### MICROCOSM EXPERIMENT

Prior to the microcosm experiment the soils were kept at 28°C for 1 week to equilibrate. Pyrene stock solutions (500, 3,000, and 7, 000 mg ⋅ L^-1^) were prepared by dissolving the chemical in acetone. Pyrene-treated soils at concentrations of 5, 30, and 70 mg ⋅ kg^-1^ *d*.*w*.*s*. were included in this microcosm experiment. The details of the soil contamination with pyrene were as follows: 25 g of soil (based on dry soil weight) were used as seedling soils for each treatment and were contaminated by spiking with 2.5 mL of the 500, 3,000, and 7,000 μg ⋅ ml^-1^ pyrene stock solutions to reach final concentrations of 50, 300, and 700 mg ⋅ kg^-1^ *d*.*w*.*s*. Soil spiked with 2.5 mL of acetone was used as seedling soil for the control (CK). All seedling soils were kept in a chemical hood overnight to allow the acetone to evaporate. The seedling soil was added to 225 g natural soil (based on dry weight) and mixed thoroughly to achieve final targeted pyrene concentrations of 5, 30, and 70 mg ⋅ kg^-1^ *d*.*w*.*s*.. After contaminating with pyrene, 10 g of soil (based on dry weight) for each treatment were aliquoted into 120 mL serum bottles; sterilized water was added to adjust the water content to 60% of the SWHC (soil water holding capacity). The bottles were capped with black butyl stoppers, and the microcosms were incubated at 28°C in darkness for 42 days. On days 0, 4, 7, 14, 21, 28, and 42, triplicate samples from each pyrene-spiked sample were collected for further pyrene analysis or DNA extraction. The headspace of the other bottles was flushed with synthetic air (20% O_2_ and 80% N_2_) for 45 s after sampling on days 7, 14, 21, 28, and 42 to maintain oxic conditions.

### PYRENE EXTRACTION AND DETERMINATION

Pyrene in soil was extracted and purified according to the method described in previous studies (Qian et al., 2007; Mao et al., 2012) with slight modifications. Specifically, soil samples were freeze-dried with a vacuum freeze drier and passed through a 0.250 mm mesh. Then, 2 g of soil was Soxhlet-extracted with 70 mL of dichloromethane for 24 h. The extract solution was rotary evaporated. The residue was dissolved in 2 mL of cyclohexane; next, 0.5 mL of the solute was transferred and purified using a silica gel column (8 × 220 mm) and washed with a mixture of hexane and dichloromethane (1:1, v/v). The first 1 mL of the eluate was discarded because it contained non-polar compounds such as saturated hydrocarbons and showed weaker retention than PAHs in silica gel. The second 2-mL aliquot of eluate was collected, evaporated to dryness under a N_2_ stream, and re-dissolved in 2 mL of acetonitrile. The solution was filtered through a 0.45 μm syringe filter membrane prior to analysis by high performance liquid chromatography (HPLC).

The analysis of pyrene concentrations was conducted on a Shimadzu Class-VP HPLC system (Shimadzu, Japan) with a fluorescence detector (RF-10AXL). The separation of pyrene was achieved using a C18 reversed phase column (VP-ODS 150 × 4.6 mm I. D., particle size 5 μm) using acetonitrile-water (4:1, v/v) as the mobile phase at a flow rate of 2 mL.min^-1^. The excitation and emission wavelengths for pyrene were 296 and 404 nm, respectively.

### DNA EXTRACTION

DNA was extracted from 0.5 g of soil collected before contamination with pyrene and the soil samples collected on day 42 using the FastDNA^TM^ spin kit for soil (MP Biomedicals LLC, OH, USA) following the manufacturer’s instructions. Briefly, cell lysis was achieved by vigorous shaking in a FastPrep®; bead-beating instrument at a speed of 6 m ⋅ s^-1^ for 40 s. The homogenized mixture was centrifuged to separate the pellet containing soil and cell debris from the DNA in the supernatant. The DNA in the supernatant was further purified and dissolved in 100 μL of elution buffer. DNA quality was assessed by electrophoresis on a 0.8% agarose gel. DNA quantity and purity were examined with a Nanodrop®; ND-1000 UV-Vis Spectrophotometer (NanoDrop Technologies, Wilmington, DE, USA). The soil DNA was stored at -20°C until use.

### REAL-TIME PCR

To determine how pyrene influences the total bacterial biomass, the real-time PCR method was used to quantify the bacterial 16S rRNA gene with a CFX96 Optical Real-Time Detection System (Bio-Rad Laboratories, Inc., Hercules, CA, USA). The assay used forward primer 515F (5′-GTGCCAGCMGCCGCGG-3′) and reverse primer 907R (5′-CCGTCAATTCMTTTRAGTTT-3′) to target the V4 region of the bacterial 16S rRNA gene. The real-time PCR standard curve for the bacterial 16S rRNA gene was generated *via* gradient dilution of plasmid DNA. Real-time PCR was performed in a 20 μL reaction mixture containing 10 μL SYBR®; *Premix Ex Taq*^TM^ (TaKaRa Biotech, Dalian, China), 0.5 μmol ⋅ L^-1^ of each primer, and 1 μL template DNA ranging from 1 to 10 ng. Amplification of the 16S rRNA gene was initiated by denaturing at 95°C for 30 s, followed by 40 cycles of 30 s at 95°C, 30 s at 55°C, 30 s at 72°C, and 30 s with a plate read. A negative control using water as a template instead of DNA was always included. Real-time PCR was performed with three technical replications for each soil DNA sample. The amplification efficiency was 106% with an *R*^2^ value of 0.994.

To quantify the abundance of key pyrene catabolic gene pyrene dioxygenase gene *nidA* that encodes the large (α) terminal dioxygenase subunit and is responsible for initial aromatic ring dihydroxylation, real-time PCR was performed in a similar manner to that used to assess 16S rRNA gene quantity with changes in the primer sequences and amplification procedure. The forward primer sequence 5′-TTCCCGAGTACGAGGGATAC and reverse primer sequence 5′-TCACGTTGATGAACGACAAA were used to target a conserved 141 bp region of the *nidA* gene (DeBruyn et al., 2007). Amplification of the *nidA* gene was performed using the following protocol (DeBruyn et al., 2007): 95°C for 30 s; 40 cycles of 94°C for 15 s, 56°C for 30 s, 72°C for 30 s, and 30 s with a plate read. The amplification efficiency was 95.3% with an *R*^2^ value of 0.999 for the *nidA* gene.

### ILLUMINA SEQUENCING AND DATA ANALYSIS

Pyrosequencing was performed on an Illumina Miseq platform by analyzing the V4 region (515F–907R) of the bacterial 16S rRNA gene. The primer 515F (5′-GTGCCAGCMGCCGCGG-3′) was fused with a 12-bp barcode (Table S1) to resolve different samples. The sequence of reverse primer 907R was 5′-CCGTCAATTCMTTTRAGTTT-3′. Each 50 μL of the PCR reaction mixture contained 1 × PCR buffer (Mg^2+^ plus), 0.2 mM of each deoxynucleoside triphosphate, 0.4 mM of each primer, 1.25 U of TaKaRa Taq HS polymerase (TaKaRa Biotech, Dalian, China), and 1 μL of soil DNA. The PCR reaction was performed in a thermal cycler (Bio-Rad Laboratories, Hercules, CA, USA) using the following cycling conditions: 94°C for 5 min, followed by 32 cycles of 94°C for 30 s, 55°C for 30 s, and 72°C for 45 s, with a final extension of 72°C for 5 min. The PCR product was visualized on a 1.8% agarose gel. The PCR amplicon band was excised from the gel and purified with an agarose gel DNA purification kit (TaKaRa Biotech, Dalian, China). The concentrations of the purified PCR amplicons were determined with a Nanodrop®; ND-1000 UV-Vis Spectrophotometer (NanoDrop Technologies, Wilmington, DE, USA); the amplicons were then pooled in equimolar concentrations into a single tube in preparation for paired-end sequencing (2 × 250 bp) using the Illumina MiSeq platform at the Chengdu Institute of Biology, Chinese Academy of Sciences.

The Illumina sequence reads were processed using the quantitative insights into microbial ecology (QIIME) pipeline ^1^. Briefly, low quality sequence reads (reads with lengths <150 bp, ambiguous bases >0, homopolymers >6, primer mismatches, and average quality scores <25) were removed and the 12-bp barcode was examined to assign the multiplexed reads to samples. Then, the Uchime algorithm was used to detect chimeric sequences with a chimera-free reference database (Edgar et al., 2011) *via* the Usearch tool. All chimeras were removed before further analysis. The clustering method was used to assign similar sequences to operational taxonomic units (OTUs) at a 97% sequence similarity level (Edgar, 2010). A representative sequence from each OTU was aligned with PyNAST (Caporaso et al., 2010). The taxonomic classification of OTUs was processed using the RDP Classifier (Wang et al., 2007). The original sequence data have been deposited in the European Nucleotide Archive under accession number PRJEB7639.

To standardize sampling efforts and bring the pyrosequence data from different samples onto a common scale, sequences were subsampled to the same sequence depth (10,000 sequences per sample) using the Perl script daisychopper.pl (Gilbert et al., 2009; Wegner et al., 2013; Li et al., 2014). The relative abundance of each group at different taxonomic levels (phylum, class, order, family, and genus) was then summarized. Principal coordinate analysis (PCoA) of the pyrosequencing data was performed using the subsampled data to determine differences in microbial community structures using weighted UniFrac distances, a measurement that accounts for the phylogenetic relationship between different sequences and thus provides far more power than the taxon-based method (Lozupone and Knight, 2005; Hamady et al., 2010). Samples were clustered based on between-samples weighted UniFrac distances using unweighted pair group method with arithmetic mean (UPGMA), and jackknifing was performed by resampling 100 times without replacement at a depth of 10,000 sequences per sample. Three different complementary non-parametric analyses for multivariate data (Zhou et al., 2012) including the analysis of similarities (ANOSIM; Clarke, 1993), non-parametric multivariate analysis of variance using distance matrices (adonis; Anderson, 2001), and a multi-response permutation procedure (MRPP; Mielke and Berry, 2001; McCune and Grace, 2002) were used to investigate community structure differences between treatments. These methods were selected because traditional multivariate statistical analyses are too stringent in their assumptions (Anderson, 2001), and traditionally, it has been difficult for all datasets to meet the assumptions (e.g., normality, equal variances, and independence) of parametric statistics (Zhou et al., 2012). Weighted UniFrac distances were used for the ANOSIM, adonis, and MRPP analyses, and a Monte Carlo permutation was exploited to test the significance of the statistics. ANOSIM, adonis, and MRPP were processed with the “vegan” package in R software version 2.15.0.

## RESULTS

### PYRENE DYNAMICS IN SOIL

**FIGURE 1:**
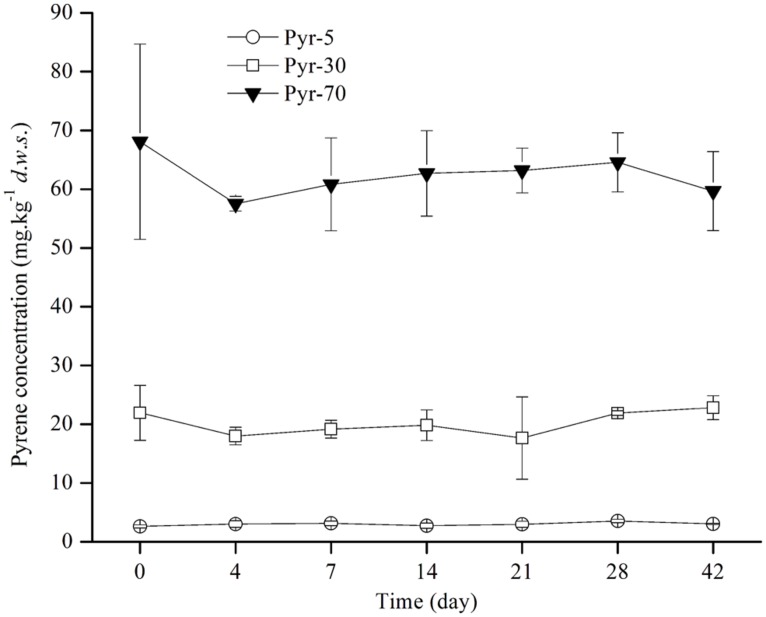
**Pyrene concentration dynamics in soil during the incubation period.** The designations Pyr-5, Pyr-30, and Pyr-70 indicate that the soil was treated with different concentrations of pyrene representing 5, 30, and 70 mg ⋅ kg^-1^ *d.w.s.*, respectively. The designation *d.w.s.* refers to dry weight soil. The error bars represent the standard deviation of the means of triplicate microcosms.

Soil microcosms were constructed with artificial pyrene contamination at three concentrations (5, 30, and 70 mg ⋅ kg^-1^ *d*.*w*.*s*) to study the biodegradation potential of red soil. The ability to degrade pyrene was investigated by detecting the concentration of pyrene on days 0, 4, 7, 14, 21, 28, and 42 during the incubation period (**Figure 1**). Intriguingly, no evident pyrene degradation was observed during the entire incubation period for all spiked pyrene concentrations.

### EFFECT OF PYRENE ON THE ABUNDANCE OF THE 16S rRNA and *nidA* GENES

**FIGURE 2:**
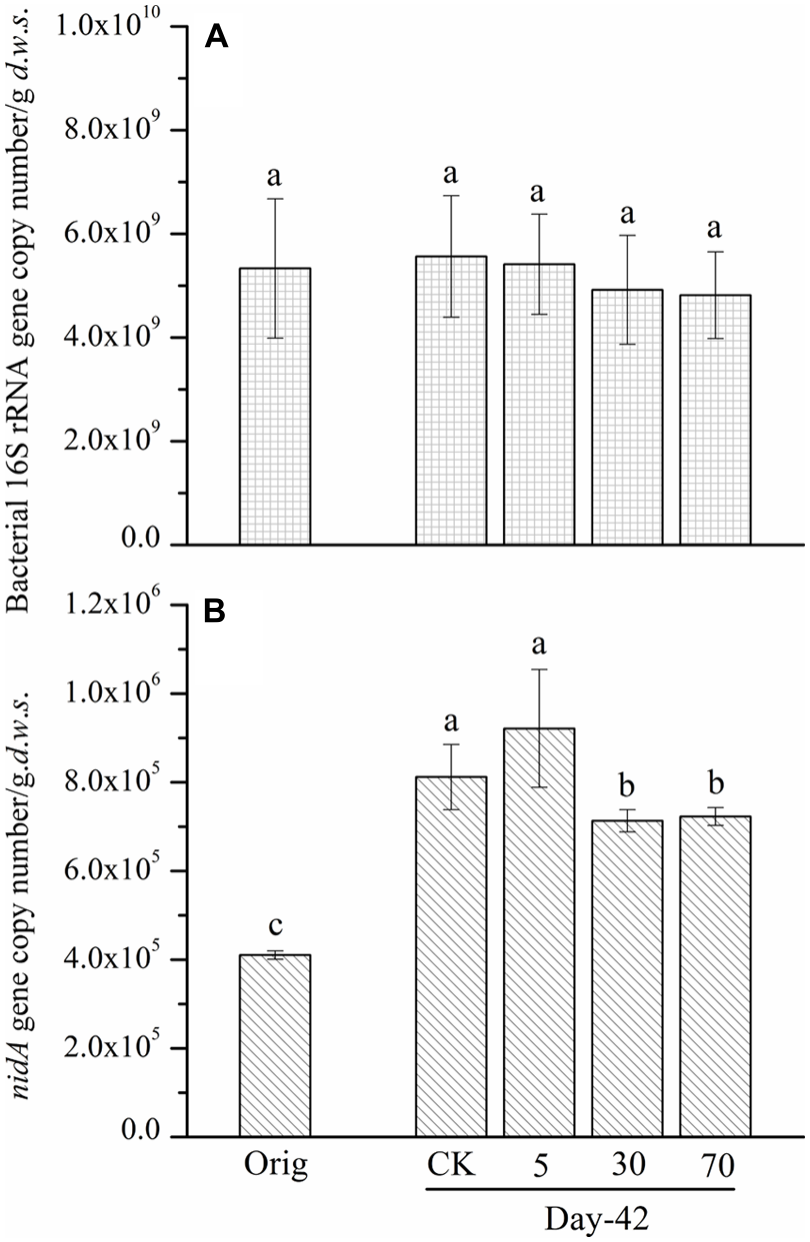
**Bacterial 16S rRNA gene **(A)** and *nidA* gene **(B)** copy number determined by real-time PCR from soil microcosms.** The designation Orig denotes the original soil that did not receive any treatment. The designation control denotes the control soil that was treated with acetone. The designations 5, 30, and 70 refer to the soils that were treated with acetone-dissolved pyrene at concentrations of 5, 30, and 70 mg ⋅ kg^-1^ *d*.*w*.*s*. Same letters indicate no significant differences by Duncan’s multiple range test (*P* < 0.05). The error bars represent the SD of the means of triplicate microcosms.

To understand the reasons that may lead to the observed slight degradation and the relationship between the abundance of pyrene-degrading bacteria and pyrene degradation, the abundance of a key pyrene-degradative gene (pyrene dioxygenase gene: *nidA*) was examined with the real-time PCR method. Additionally, the 16S rRNA gene and the soil DNA content were quantified as a proxy for bacterial biomass. After 42 days of incubation, the abundance of the 16S rRNA gene copy number (biomass) in the control soil was unchanged, 5.56 × 10^9^ copies per gram dry weight soil, compared with the original soil, 5.33 × 10^9^ (**Figure 2A**). The persistence of pyrene to degradation at all spiking concentrations led to a non-significant (*P* > 0.05) decrease in 16S rRNA gene copy number (biomass; **Figure 2A**). A similar pattern was observed for DNA content (Figure S1). Additionally, after 42 days of incubation, the quantity of the *ndiA* gene in the soils spiked with the tested low concentration of pyrene (5 mg ⋅ kg^-1^ *d*.*w*.*s*.) was not significantly affected compared with that of the control soil (**Figure 2B**). Interestingly, when the pyrene spiking concentration was increased to 30 mg ⋅ kg^-1^ *d*.*w*.*s*., the quantity of the *nidA* gene was significantly decreased (*P* < 0.05) compared with the control (**Figure 2B**). When the pyrene spiking concentration increased to 70 mg ⋅ kg^-1^ *d*.*w*.*s*., a further significant decrease in *nidA* gene copy number was observed (**Figure 2B**).

### EFFECT OF PYRENE ON BACTERIAL RICHNESS AND DIVERSITY

The Illumina sequencing data were used to evaluate the effect of the presence of pyrene on bacterial richness and diversity. Based on the Illumina data, a total of 425,672 high quality sequences (Table S2) across all 15 samples or an average of 28,378 sequences for each sample were obtained after applying all quality filters. A total of 91.4% of the sequences were taxonomically classified as bacteria, and only 0.41% of the sequences were identified as archaea (Table S2).The observed OTUs (Figure S2A) and Shannon indices (Figure S2B) were used to determine whether the presence of pyrene resulted in adverse effects on bacterial richness and diversity. Pyrene addition resulted in a slight significant decrease in the number of OTUs and the Shannon index compared with the control treatment after 42 days of incubation. The higher the concentration of the spiked pyrene, the lower the bacterial richness and the diversity became.

### EFFECT OF PYRENE ON THE BACTERIAL COMMUNITY STRUCTURE

**FIGURE 3:**
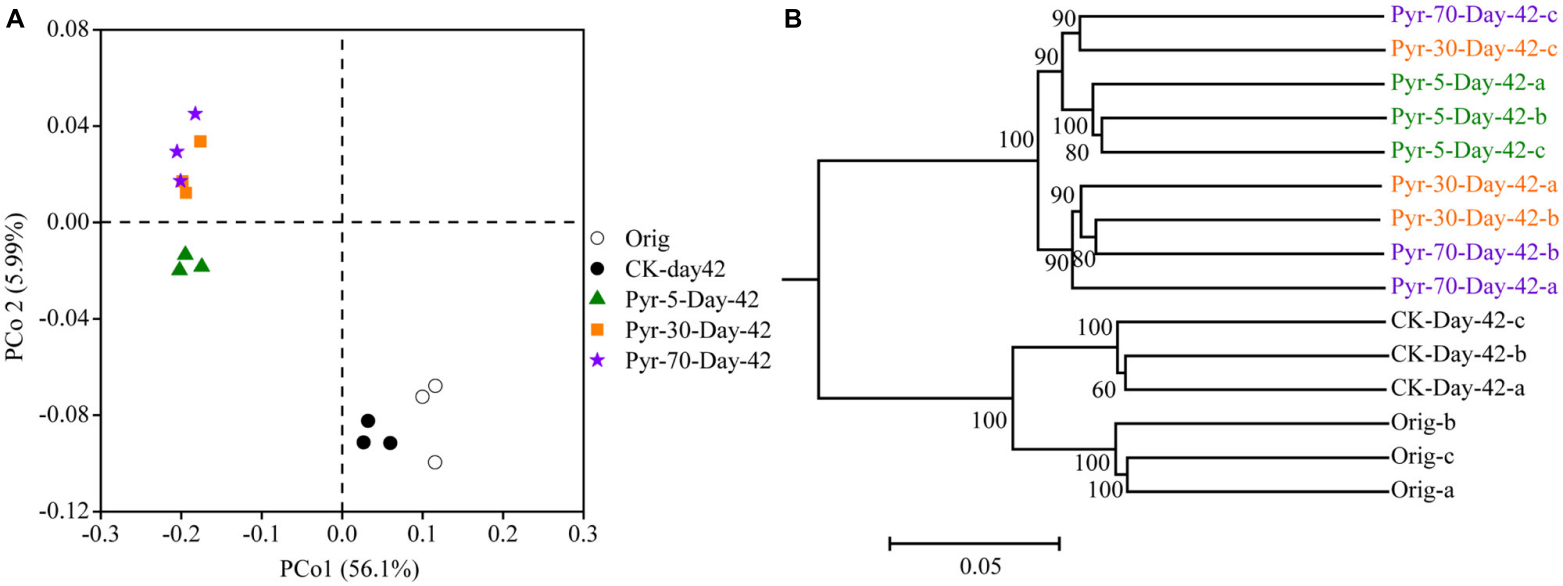
**Principal coordinate analysis (PCoA) **(A)** and unweighted pair group method with arithmetic mean cluster analysis **(B)** of microbial communities based on weighted UniFrac distances at a depth of 10,000 randomly sampled sequences per sample.** The percentages in parentheses indicate the proportions of variation explained by each ordination axis. The numbers on the nodes indicate bootstrapping values for each node. The symbols “a”, “b”, and “c” near “Day-42” or “Orig” indicate triplicate microcosms for each treatment. All other designations are the same as those in **Figure 2**.

The bacterial community structure is an overall reflection of the composition and abundance of different bacterial phylotypes within a microbial community. Therefore, changes in community structure were analyzed using the PCoA and UPGMA methods to evaluate specific effects of pyrene addition on the bacterial community from the viewpoint of microbial ecology. The day 42 samples from the control (CK) group clustered with the original soil samples in the PCoA data space (**Figure 3A**) and the UPGMA tree (**Figure 3B**), suggesting similar microbial community structures in the pyrene un-amended soil during the incubation period. Intriguingly, after 42 days of incubation, the pyrene-spiked samples formed a distinct cluster distant from the original soil samples and pyrene un-amended soil samples collected on day 42. Three non-parametric multivariate statistical tests (ANOSIM, adonis, and MRPP) also showed significant differences (*P* < 0.05) between the pyrene-spiked samples and the control samples collected on day 42 (**Table 1**). Additionally, within the pyrene-spiked soils the samples were generally further separated (**Figure 3A**) and clustered (**Figure 3B**) by pyrene dosage; this result indicated that the pyrene levels in soil resulted in difference in microbial community structures.

**Table 1:** Significance tests using three statistical approaches to assess the effects of pyrene on overall microbial community structure on day 42.

Compared groups	adonis^a^		ANOSIM^b^		MRPP^c^	
	*F*	*P*^d^	*R*	*P*^d^	*δ*	*P*^d^
CK-Day-42 vs. Pyr-5-Day-42	25.293	0.038	1	0.039	0.231	0.039
CK-Day-42 vs. Pyr-30-Day-42	23.026	0.036	1	0.036	0.249	0.035
CK-Day-42 vs. Pyr-70-Day-42	27.249	0.038	1	0.042	0.248	0.045

*All tests (adonis, ANOSIM, and MRPP) are non-parametric multivariate analyses based on dissimilarities among samples*.

*^a^Permutational multivariate analysis of variance using distance matrices. The significance was determined by *F*-tests based on sequential sums of squares from permutations of the raw data*.

*^b^Analysis of similarities. The *R* statistic was obtained based on the difference of the mean ranks between groups and within groups. The statistical significance of the observed *R* was tested by permuting the grouping vector to obtain the empirical distribution of *R* under the null-model*.

*^c^Multi-response permutation procedure. The MRPP statistic *δ* is the overall weighted mean of the within-group means of the pairwise dissimilarities among sampling units. The significance tests indicate the fraction of permuted δ that is less than the observed δ*.

*^d^The Bonferroni correction for the *P* value of the significance test was used*.

*All other designations are the same as those in **Figure 2***.

### EFFECT OF PYRENE ON SPECIFIC BACTERIAL POPULATIONS AT THE HIGH TAXONOMIC LEVEL

**FIGURE 4:**
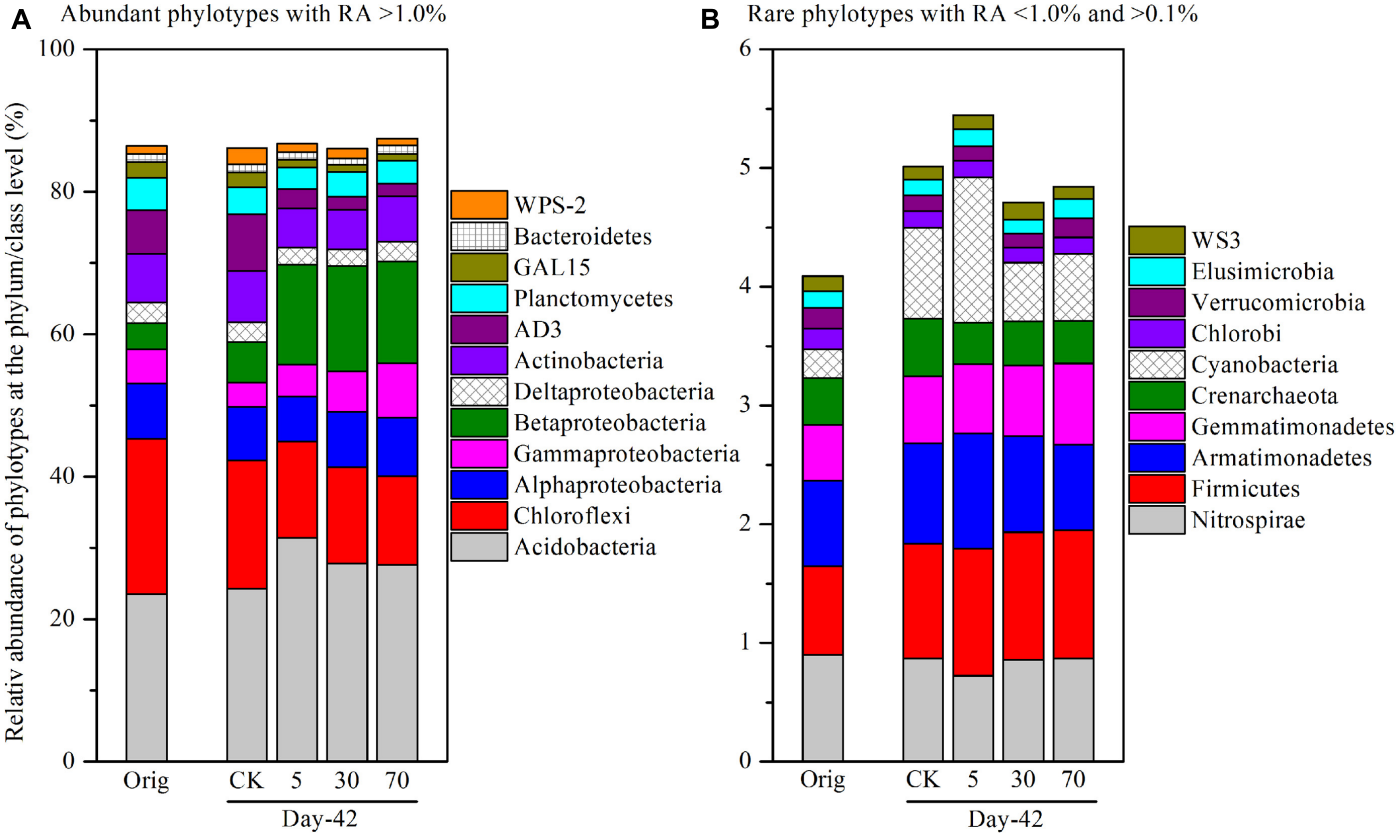
**Relative abundance of abundant phylotypes **(A)** and rare phylotypes **(B)** at the phylum or class (only for Proteobacteria) level.** Abundant phylotypes refer to those microbial groups with relative abundance (RA) >1.0% under all treatments. Rare phylotypes refer to those microbial group with RA <1.0% under at least one treatment and >0.1% under all other treatments. The statistical analysis results are the same as those in **Figure 6**. All other designations are the same as those in **Figure 2**.

To identify specific bacterial populations that may be affected by pyrene, the relative abundance of the phylotypes were summarized at the phylum or lower taxonomic levels. The shift in relative abundance of the main phyla/classes (relative abundance >0.1% were included) under different treatments is shown in **Figure 4A**. Although the relative abundance of the main phyla varied greatly under different treatments, 12 phyla/classes including Acidobacteria (23.6–31.4%), Chloroflexi (12.4–21.8%), Alphaproteobacteria (6.36–8.21%), Betaproteobacteria (3.60–14.8%), Gammaproteobacteria (3.42–7.70%), Deltaproteobacteria (2.30–2.97%), Actinobacteria (5.44–7.22%), AD3 (1.74–7.95%), Planctomycetes (3.09–4.60%), GAL15 (1.00–2.20%), Bacteroidetes (1.00–1.26%), and WPS-2 (1.00–2.28%) were always abundant (relative abundance >1.0% in all treatments) and shared bacterial groups (**Figure 4A**), accounting for a total of 86.0–87.5% of the whole community. Rare phyla (relative abundance <1.0% in at least one treatment and >0.1% in all treatments) comprised 10 phyla (**Figure 4B**) including Nitrospirae, Firmicutes, Armatimonadetes, Gemmatimonadetes, Crenarchaeota, Cyanobacteria, Chlorobi, Verrucomicrobia, Elusimicrobia, and WS3, accounting for a total of 4.09–5.45% of the whole community.

**FIGURE 5:**
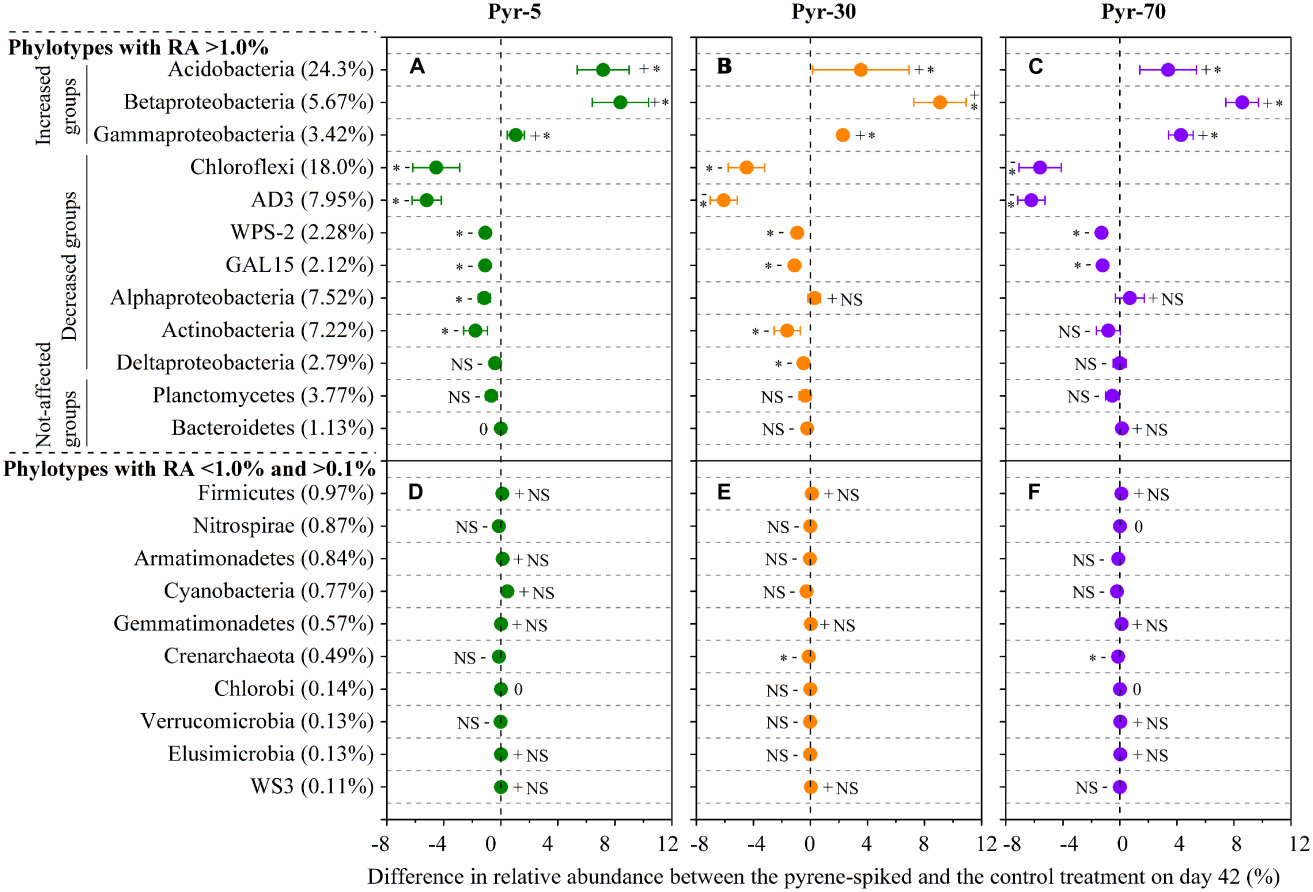
**Differences in relative abundance between the pyrene-spiked and control microcosms on day 42 for the abundant (relative abundance >1.0% in all treatments) **(A–C)** and rare (relative abundance <1.0% in at least one treatment and >0.1% in all treatments) **(D–F)** phylotypes at the phylum or class (only for Proteobacteria) level.** The net difference in relative abundance was calculated as the relative abundance under pyrene-spiked treatment – the relative abundance of the phylotype under control treatment. The error bars represent the standard error of the means. As for the abundant phylotypes, groups increased in relative abundance (Increased groups) are shown first, followed by phylotypes that are decreased (Decreased groups) in relative abundance and phylotypes that are not significantly affected by pyrene (Not-affected groups). For the rare phylotypes, the microbial populations are presented in descending order on the basis of their relative abundance in the control treatment because only one population was significantly affected by pyrene. The percent value in the parentheses refers to the relative abundance in the control treatment. The symbols “+”, “-”, and “0” indicate that the relative abundance was increased, decreased, or stable compared with the control. The symbol “*” denotes significant differences at *P* < 0.05; NS denotes no significant difference (*P* > 0.05). All other designations are the same as those in **Figure 2**.

As total bacterial biomass was not significantly influenced by pyrene (**Figure 2A**; Figure S1), changes in relative abundance could then reflect the absolute abundance of specific phylotypes. It is expected that the persistence of pyrene to degradation may exert a toxic influence on some bacterial populations because the bacterial richness (Figure S2A) and diversity (Figure S2B) were decreased by pyrene. Indeed, seven out of the 12 abundant phyla/classes were significantly (*P* < 0.05) decreased in relative abundance with at least one concentration of pyrene after 42 days of incubation (**Figure 5A–C**). Specifically, Chloroflexi, AD3, WPS-2, and GAL15 were significantly decreased by all the tested pyrene dosages, including 5, 30, and 70 mg ⋅ kg^-1^, Actinobacteria was significantly reduced at concentrations of 5 and 30 mg ⋅ kg^-1^, and the Alphaproteobacteria and Deltaproteobacteria were significantly decreased at 5 and 30 mg ⋅ kg^-1^, respectively. Additionally, the phylum Crenarchaeota (belong to Archaea) within the rare group was also significantly decreased (*P* < 0.05) by pyrene at a concentration of 30 mg ⋅ kg^-1^ (**Figure 5E**). However, we did not observe a significant effect of pyrene on the relative abundance of the abundant groups Planctomycetes and Bacteroidetes (**Figure 5A–C**) and the nine other rare phylotypes including Firmicutes, Nitrospirae, Armatimonadetes, Cyanobacteria, Gemmatimonadetes, Chlorobi, Verrucomicrobia, Elusimicrobia, and WS3 (**Figure 5D–F**). Intriguingly, some phylotypes including Acidobacteria, Betaproteobacteria, and Gammaproteobacteria were enriched, as revealed by the significant increase in relative abundance at different concentrations of pyrene (**Figure 5A–C**) although significant pyrene degradation was not observed at any pyrene concentration (**Figure 1**). These results indicate that the persistence of pyrene to degradation affected the abundance of specific populations at a higher taxonomic level (phyla/classes level).

### EFFECT OF PYRENE ON SPECIFIC BACTERIAL POPULATIONS AT THE LOWER TAXONOMIC LEVEL

**FIGURE 6:**
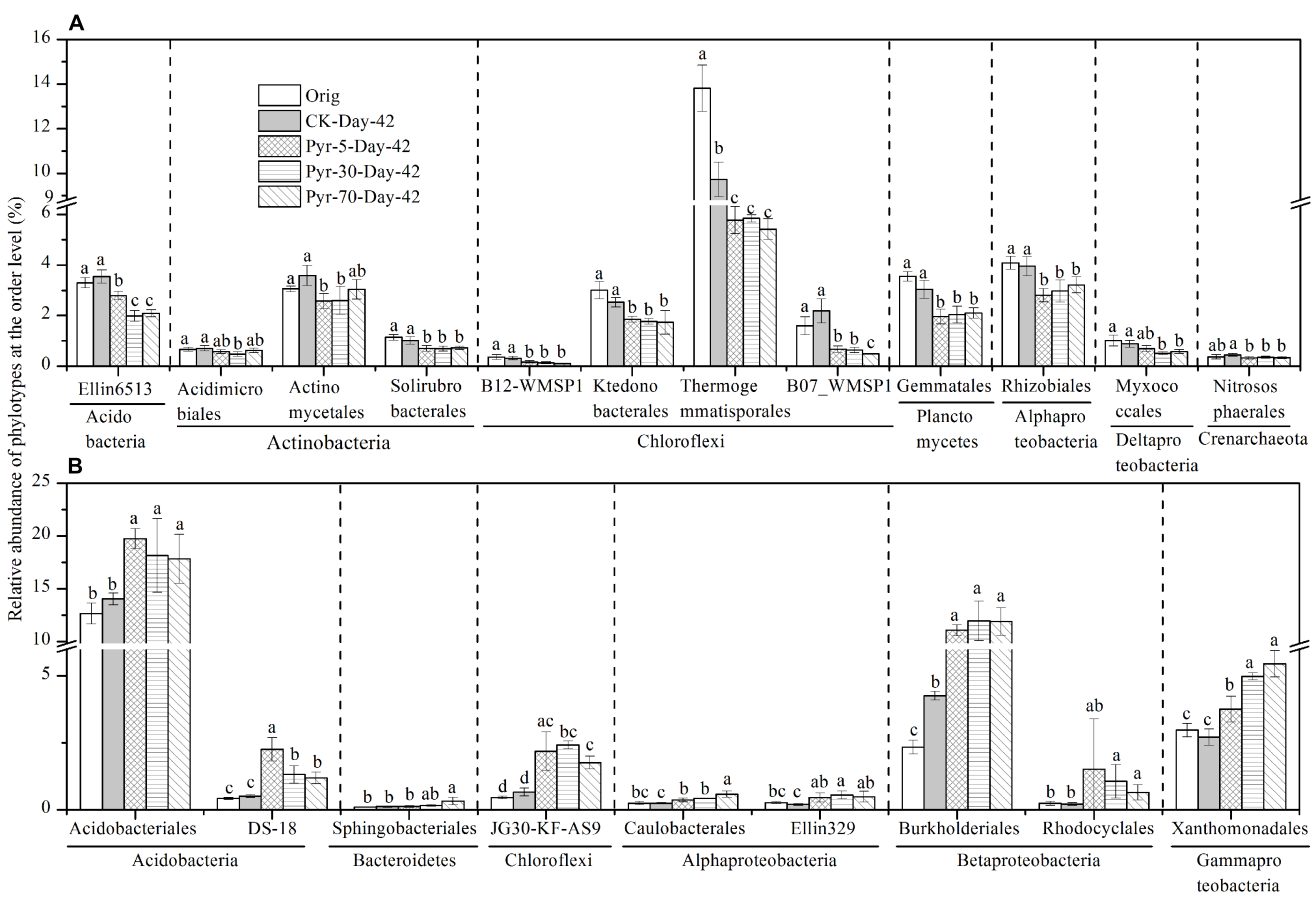
**Effect of pyrene on specific bacterial populations at lower taxonomic level (order level).** Only those phylotypes which could be classified at the order level and had relative abundance >0.1% are presented. **(A)** Phylotypes that were decreased in relative abundance by pyrene spiking. **(B)** Phylotypes that were increased in relative abundance by pyrene spiking. Different letters indicate significant differences by Duncan’s multiple range test (*P* < 0.05). The error bars represent the standard deviation of the means of triplicate microcosms. All other designations are the same as those in **Figure 2**.

More than 88.9% of the sequences could be taxonomically classified at the taxonomic level of order or higher, but only 62.6 and 17.7% of the sequences could be classified at the family and genus levels, respectively (Table S2). The relative abundance of each phylotype was then further analyzed at the order level to understand which bacterial populations were affected by pyrene on a finer taxonomic level. Among the main orders (with relative abundance >0.1%), those phylotypes that were significantly affected by pyrene in terms of relative abundance and could be taxonomically classified at the order level are shown in **Figure 6**. Twelve orders (**Figure 6A**) covering the phyla Acidobacteria, Actinobacteria, Chloroflexi, Planctomycetes, Alphaproteobacteria, Deltaproteobacteria, and Crenarchaeota were significantly reduced (*P* < 0.05) in relative abundance following incubation with at least one concentration of pyrene. Intriguingly, nine orders were found to be increased in relative abundance (**Figure 6B**) although no significant pyrene degradation was observed. These results further revealed that pyrene affected specific microbial populations at a lower taxonomic rank (order level).

## DISCUSSION

We did not observe evidence of pyrene degradation in the tested red soil. To some extent, the following reasons may explain this phenomenon.

According to our Miseq sequencing data, some previously reported pyrene-degradative phylotypes were detected in the soil, but their relative abundance were reduced or remained stable in the presence of pyrene. The genus *Mycobacterium* is a widely distributed (DeBruyn et al., 2009) and well characterized (Stingley et al., 2004; Kim et al., 2008; DeBruyn et al., 2011) pyrene-degradative population. A few studies have found that the abundance of fast-growing *Mycobacterium* was positively associated with pyrene degradation (DeBruyn et al., 2007; Peng et al., 2010). Interestingly, our study revealed that the relative abundance of *Mycobacterium* following the pyrene-spiking treatment was significantly decreased (*P* < 0.05) compared with the control treatment (Figure S3A) after 42 days of incubation. Four other bacterial phylotypes found to be able to degrade pyrene in a small number of studies including *Bacillus* (Ling et al., 2011), *Rhodococcus* (Walter et al., 1991), *Pseudoxanthomonas* (Klankeo et al., 2009), and *Microbacterium* (Wongwongsee et al., 2013) were also detected in our study. However, their relative abundance remained stable following exposure to pyrene after a 42-day incubation period (Figures S3B–E). These results revealed toxic effect of pyrene on some functional groups.

A degradative population is usually considered to possess key degradative genes such as the PAH dioxygenase gene (DeBruyn et al., 2007, 2009). Some studies have reported positive relationships between PAH biodegradation/contamination and the abundance of PAH-dioxygenase genes (Dionisi et al., 2004; Tuomi et al., 2004). Peng et al. (2010) found that the copy number of pyrene dioxygenase gene (*nidA* gene) increased to approximate 500-fold in a pyrene polluted soil and the dissipation of pyrene was up to 80% after 2 months of incubation. Therefore, the abundance of *nidA* gene was considered to serve as the biomarker for pyrene degradation (DeBruyn et al., 2007; Peng et al., 2010). In our study, real-time PCR data showed stability with regard to 16S rRNA gene copy number after pyrene exposure; microbial DNA content was also unaffected by pyrene. However, a significant decrease (at 30 and 70 mg ⋅ kg^-1^ *d*.*w*.*s* of pyrene) in the pyrene dioxygenase gene (*nidA*) was observed. These results demonstrate that existence of pyrene exerted a deleterious influence on specific bacterial populations such as pyrene-degrading phylotypes. On the other hand, the significant decrease or stability of the abundance of *nidA* gene also demonstrates that Gram-positive bacteria were not able to degrade pyrene in the tested soil since *nidA* is usually found in Gram-positive bacteria like Actinobacteria (Khan et al., 2001; Brezna et al., 2003; Cébron et al., 2008; DeBruyn et al., 2009). This was supported by our results which showed that the relative abundance of Actinobacteria was reduced. It should be noted that the primers used for amplify the *nidA* gene were designed according to determination of the conserved regions of *nidA* from several pyrene-degrading *Mycobacterium* (DeBruyn et al., 2007). Designing a more universal primer targeting pyrene dioxygenase genes from more pyrene-degrading bacterial populations rather than just from *Mycobacterium* may be helpful for further study.

Furthermore, some putative pyrene degrading bacteria may not be at work in pyrene degradation in the tested soil. The exposure time to pyrene could be considered as one of limited factors. The growth, multiplication, and adaptation of the pyrene-degradative populations may be a time-consuming process and the 42 days of exposure to pyrene may not be long enough to lead to the formation of the pyrene-degradative consortium. Johnsen and Karlson (2005) found that pyrene could be mineralized in soils collected from industrialized areas rather than in pristine forest soils within a 140-day test period. Additionally, it is also possible that the dosage of pyrene is not high enough since different microbes have catabolic enzymes that are activated at different levels of pollution. A previous study has shown that only when the concentration of naphthalene was higher than 30 μM could the *Pseudomonas* isolates actively degrade naphthalene (Huang et al., 2009). Furthermore, some other environmental factors, such as the molar ratio of carbon, nitrogen, and phosphate (C/N/P), the nitrogen form, pH, etc., may limit the PAH degradation (Roling et al., 2002; Simarro et al., 2011). Simarro et al. (2011) have optimized the six key abiotic factors in the process of PAH (naphthalene, phenanthrene, and anthracene) biodegradation by a bacterial consortium and revealed that high concentrations of nutrients (C/N/P molar ratio of 100:21:16; a mixture of glucose and PAHs as carbon source) and soluble forms of nitrogen and iron (NaNO_3_ as nitrogen source; Fe_2_(SO_4_)_3_ as iron source using a concentration of 0.1 mol ⋅ L^-1^) at neutral pH (7.0) favored the biodegradation. Therefore, further multifactorial experiment is necessary to determine the constraint abiotic factors leading to the non-degradation of pyrene in the red soil.

Pyrene addition slightly lowered the bacterial richness and diversity and altered the bacterial community structure. In total, within taxonomically classified phylotypes at the phylum or class (only for Proteobacteria) level, the relative abundance of seven abundant phyla/classes (relative abundance >1.0%) and one phylum with relatively low abundance (relative abundance <1.0 and >0.1%) were significantly reduced by pyrene treatment at a coarse taxonomic level. Additionally, 12 orders were decreased in relative abundance at a finer taxonomic level. A total of three phyla/classes and nine orders were significantly increased in relative abundance (termed as increased phylotypes), possibly as a result of improved competitive ability. We speculate that there may be niche competition (Cunliffe and Kertesz, 2006) between the increased and decreased populations. The increased phylotypes might possess ecological advantages that allow them to survive, multiply, and compete in contaminated soils. It is also possible that the decreased phylotypes were more sensitive to pyrene, resulting in cell death due to pyrene toxicity. In this case, nutrients released from the cells of the decreased phylotypes after death could be utilized by the increased phylotypes for better growth. Further work should be performed to test this hypothesis.

Several populations were significantly decreased in relative abundance such as Actinobacteria (including three orders, Acidimicrobiales, Actinomycetales, and Solirubrobacterales). The decrease of Actinobacteria is contrast with most studies which found that the phylum was enriched where PAH degradation was observed based on the 454 pyrosequencing method (Sun et al., 2014) or stable isotope probing technique (Peng et al., 2013). Lower relative abundance of Actinobacteria in high PAH pollution has very recently been reported (Mukherjee et al., 2014). Moreover, some microbial taxa were indeed enriched at the phylum, class, or order level. Four significantly enriched orders were observed, including Burkholderiales and Rhodocyclales in Betaproteobacteria, Xanthomonadales in Gammaproteobacteria, and Sphingobacteriales in Bacteroidetes. These groups have been found in other soils or sediments contaminated by petroleum hydrocarbon, PAHs, and other hydrocarbons (Gomes et al., 2010; Greer, 2010; Mao et al., 2012; Castro-Silva et al., 2013), but in our case were not able to degrade the added pyrene.

In summary, the biodegradation of pyrene was not observed when the tested clean soil (not a PAH historically contaminated soil) was spiked with pyrene. The poor degradation ability was associated with the stability or significant decrease of the abundance of the pyrene dioxygenase gene (*nidA*). Even though some well-known pyrene-degrading populations such as *Mycobacteirum*, *Bacillus*, *Rhodococcus*, *Pseudoxanthomonas*, and *Microbacterium* were detected in the soil according to the Miseq data, they were not at work in pyrene degradation based on the observation that their relative abundance was reduced or stable. Although not degraded, pyrene led to a slight decrease in bacterial richness after 42 days of incubation and altered the whole bacterial community structure. This work is helpful for the ecological evaluation of the effect of PAHs on soil ecosystems in terms of microbial ecology.

## Conflict of Interest Statement

The authors declare that the research was conducted in the absence of any commercial or financial relationships that could be construed as a potential conflict of interest.

## Footnotes

1 http://qiime.org/tutorials/tutorial.html
