# Supplementary material for: Evident bacterial community changes but only slight degradation when polluted with pyrene in a red soil
Source: Front Microbiol. 2015 Jan 30;6:22. doi: 10.3389/fmicb.2015.00022 (PMC4311681; doi:10.3389/fmicb.2015.00022)
Supplement: Supplementary file 1 [file Presentation_1.PDF]

# *Supplementary Material*

# Evident bacterial community changes but only slight degradation when polluted with pyrene in a red soil

**Gaidi Ren[1], Wenjie Ren[1], Ying Teng[1*], Zhengao Li[1]**

[1] Key Laboratory of Soil Environment and Pollution Remediation, Institute of Soil Science, Chinese Academy of Sciences, Nanjing, China

*** Correspondence:** Ying Teng, Key Laboratory of Soil Environment and Pollution Remediation, Institute of Soil Science, Chinese Academy of Sciences, East Beijing Road No. 71, Nanjing, Jiangsu Province, 210008, China

yteng@issas. ac.cn

**This file includes:**

Supplementary Table S1 and S2

Supplementary Figure S1, S2, and S3

**Supplementary Tables**

Table S1. Barcode sequence information for each sample.

| Sample | Barcode sequence |
|---|---|
| Orig-a | GTAGTGTCAACA |
| Orig-b | GACCCTAGACCT |
| Orig-c | TATTCAGCGGAC |
| CK-Day-42-a | ACCGTCTTTCTC |
| CK-Day-42-b | AGTCTGTCTGCG |
| CK-Day-42-c | CCGCACTCAAGT |
| Pyr-5-Day-42-a | CGTATAAATGCG |
| Pyr-5-Day-42-b | AATACAGACCTG |
| Pyr-5-Day-42-c | TAAGACTACTGG |
| Pyr-30-Day-42-a | CGCGAAGTTTCA |
| Pyr-30-Day-42-b | CGATACACTGCC |
| Pyr-30-Day-42-c | TTGAAATCCCGG |
| Pyr-70-Day-42-a | GTTAGGGAGCGA |
| Pyr-70-Day-42-b | TTACTGTGGCCG |
| Pyr-70-Day-42-c | ATATAAGGCCCA |

The designation Orig denotes the original soil that did not receive any treatment. The designation CK denotes the control soil that was treated with acetone. The designations Pyr-5, Pyr-30, and Pyr-70 refer to the soils that were treated with acetone-dissolved pyrene at concentrations of 5, 30, and 70 mg.kg$^{-1}$ *d.w.s.*, respectively. The designation Day-42 denotes the soil microcosm incubated for 42 days. The symbols “a”, “b”, and “c” near “Day-42” or “Orig” indicate triplicate microcosms for each treatment.

Table S2. High quality sequence numbers and percentage of sequences classified at different taxonomic levels.

| Sample | High quality sequence numbers | Percentages of bacteria in whole community | Percentages of archaea in whole community | Percentages of sequences classified at different taxonomic levels (%) | | | | |
|---|---|---|---|---|---|---|---|---|
| | | | | Phylum | Class | Order | Family | Genus |
| Orig | 31,843±13,120 | 90.4±1.4 | 0.40±0.06 | 90.8±1.4 | 87.0±1.2 | 87.7±0.5 | 61.0±1.1 | 11.1±0.2 |
| CK-Day-42 | 41,374±15,930 | 90.7±0.7 | 0.39±0.04 | 91.0±0.8 | 86.4±0.9 | 84.3±1.0 | 57.2±1.3 | 12.6±0.3 |
| Pyr-5-Day-42 | 26,184±3,662 | 92.1±0.6 | 0.47±0.09 | 92.6±0.6 | 90.2±0.5 | 91.0±1.0 | 64.5±2.0 | 20.8±0.9 |
| Pyr-30-Day-42 | 17,735±9,493 | 90.8±0.3 | 0.38±0.01 | 91.1±0.4 | 88.6±0.2 | 92.6±0.4 | 66.2±0.5 | 24.8±1.6 |
| Pyr-70-Day-42 | 24,755±10,666 | 92.6±0.7 | 0.43±0.06 | 93.0±0.7 | 91.0±0.5 | 93.0±0.5 | 68.4±1.5 | 25.8±0.9 |
| Total | 425,672 | 91.4 | 0.41 | 91.8 | 88.5 | 88.9 | 62.6 | 17.7 |

The data presented are the mean value of triplicate microcosms ± the standard deviation. All other designations are the same as those in Table S1.

**Supplementary Figures**

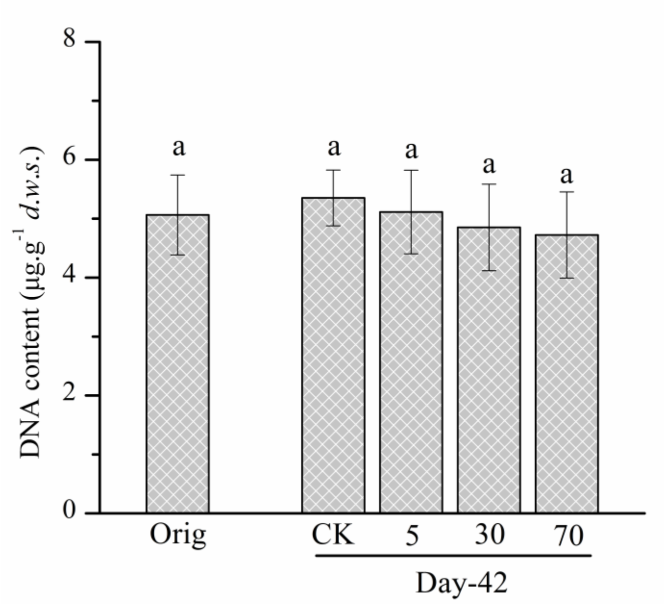


Figure S1. Soil DNA content. The data shown are the mean value of triplicate microcosms and the standard deviation. Different letters indicate significant differences by Duncan's multiple range test (*P*<0.05). All other designations are the same as those in Table S1.

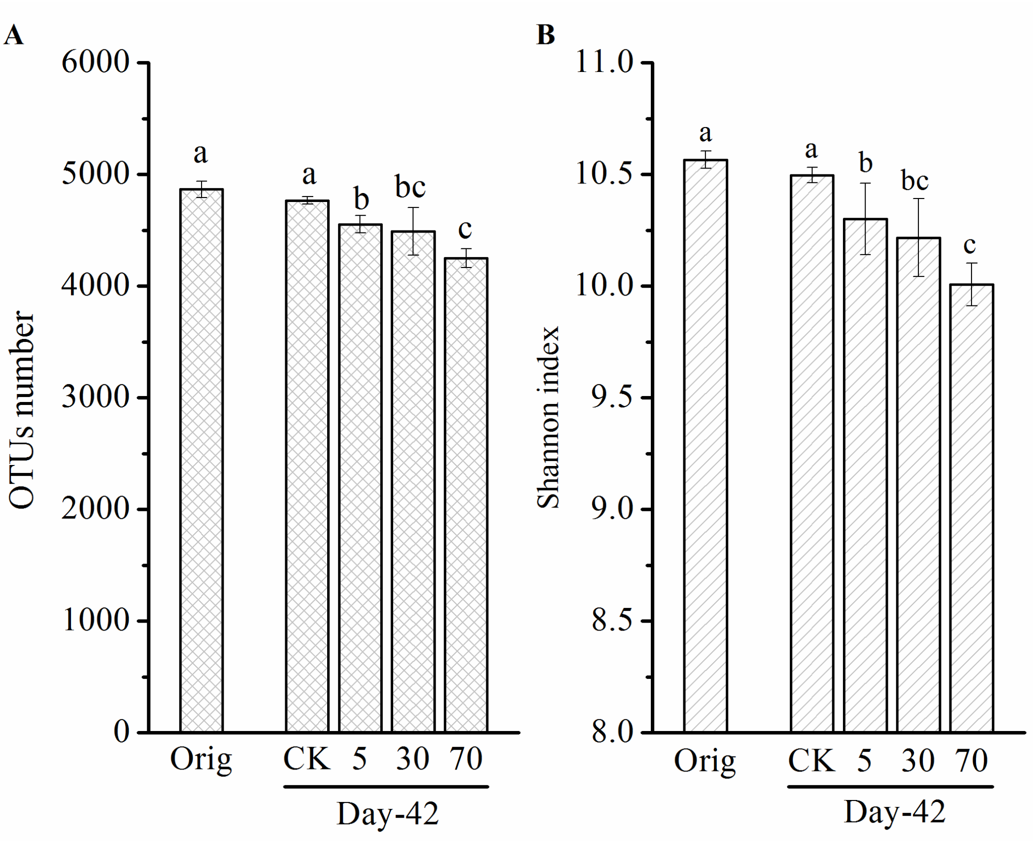


Figure S2. Observed operational taxonomic units (OTUs) numbers and Shannon index. The OTU (on 97% similarity basis) number and Shannon index were calculated for a randomized subset of 10,000 reads per sample. Different letters indicate significant differences by Duncan's multiple range test ($P$<0.05). The error bars represent the standard deviation of the means of triplicate microcosms. All other designations are the same as those in Table S1.

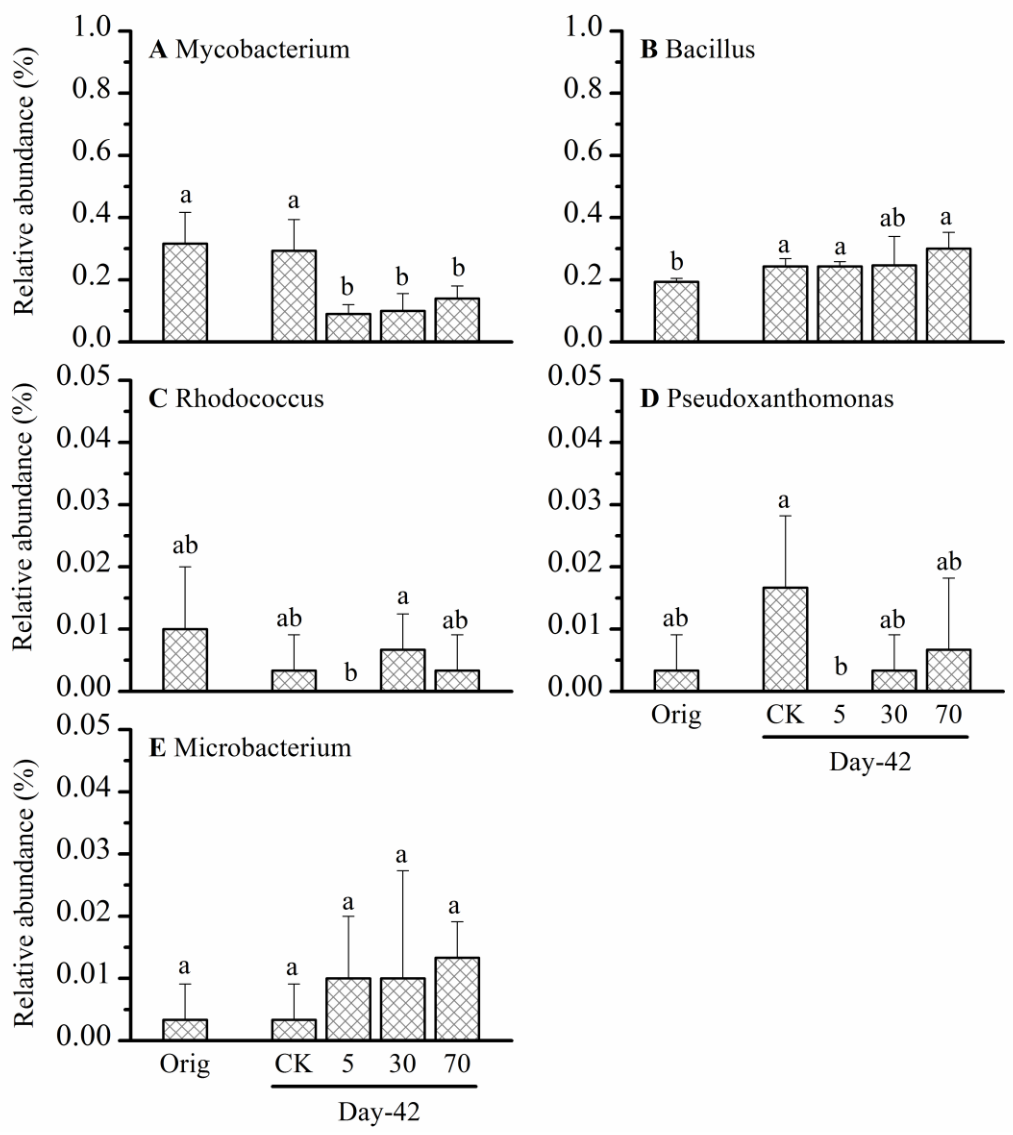


Figure S3. Relative abundance of different genera. Genera reported to be able to degrade pyrene are included. The data shown are the mean value of triplicate microcosms and the standard deviation. Different letters indicate significant differences by Duncan's multiple range test ($P$<0.05). All other designations are the same as those in Table S1.
